# Chitosan-Gentamicin Conjugate Hydrogel Promoting Skin Scald Repair

**DOI:** 10.3390/md18050233

**Published:** 2020-04-29

**Authors:** Tingting Yan, Songzhi Kong, Qianqian Ouyang, Chengpeng Li, Tingting Hou, Yu Chen, Sidong Li

**Affiliations:** 1 Faculty of Chemistry and Environmental Science, Guangdong Ocean University, Zhanjiang 524088, China; 13922085100@163.com (T.Y.); ouyangqianqian0426@163.com (Q.O.); htt0415@126.com (T.H.); 13702737491@163.com (S.L.); 2 College of food science and technology, Yunnan Agricultural University, Kunming 650000, China; 3 School of Material Science and Engineering, Beijing Institute of Technology, Beijing 100081, China; bityuchen@bit.edu.cn

**Keywords:** chitosan-gentamicin conjugate, antimicrobial, anti-inflammatory, scald repair

## Abstract

Our earlier research indicated that chitosan-gentamicin conjugate (CS-GT) possesses superior antimicrobial activity and good water solubility. To develop CS-GT-based scald dressings, the antibacterial properties of CS-GT were further studied, and the biosafety of CS-GT and the healing mechanism of CS-GT hydrogel was systematically explored in this article. It was found that cell viability shows a declined inclination with the prolonged culture time and the increased concentration of CS-GT. After three day’s culture, the cell viability could still remain at 79.72% when CS-GT concentration was as high as 1000 μg/mL. On the other hand, the hemolysis rate of CS-GT was lower than 5% when its concentration is 800 μg/mL. Therefore CS-GT has good cytocompatibility and hemocompatibility. A wound-healing experiment has shown that the skin healing rate of CS-GT hydrogel was the highest at 99.61%, followed by the positive control (wet burn ointment) 94.98%, GT hydrogel 87.50%, and matrix 77.39%. The blank control group, however, possessed the lowest healing rate of 75.45%. Further analysis indicated that CS-GT hydrogel could promote the synthesis of total protein (TP) in skin granulation tissue, resulting in the enhanced hydroxyproline (HYP) content, which facilitated collagen fibrogenesis, reduced cytokine expression in an inflammatory response, and, ultimately, accelerated wound healing. To sum up, CS-GT hydrogel is a promising scald dressing.

## 1. Introduction

Skin is the first line of human body defense against ambiance, featuring functions such as resisting microbial invasion, maintaining body fluid and water equilibrium, and regulating body temperature [1]. Upon scalding, the first line of defense is destroyed, and wound exudates such as protein and necrosed tissue prosper at the scald site, offering adequate high-nutrient substances for microbial growth and reproduction, which enables higher wound infection rates and is highly prone to an intense inflammatory response [2]. The inflammation phase of skin tissue regeneration is a crucial phase of normal wound healing, characterized by continuous infiltration of neutrophils, macrophages, and lymphocytes [3]. A few minutes after getting hurt, neutrophils arrive at the wound site and start action for several days, and they themselves are phagocytosed by tissue macrophages. Although the primary role of neutrophils is to kill bacteria, neutrophils are also a source of proinflammatory cytokines including interleukins 1α and β (IL-1α and IL-β) and tumor necrosis factor α (TNF-α) [4]. Providing the earliest signal to inactivate fibroblasts and keratinocytes at the wound [5], these proinflammatory cytokines play a very important role in wound healing. Moreover, in the presence of microorganisms, free radicals and reactive oxygen species released at the wound site during this phase incur serious complications including infection [6], delayed healing process, and serious wound dehydration [7], while dehydration interrupts desirable moist healing environment and further postpones wound healing. Even though considerable progress has been made in burn care and treatment nowadays, infections are still an overarching risk of increased patient deaths [8]. Therefore, a desirable dressing for trauma repair should be nontoxic, non-adherent, capable of absorbing excessive exudate, and have a number of excellent properties such as efficient microbial resistance and biocompatibility.

In recent years, biomaterial-based wound dressings have been widely used, such as chitosan (CS) [9], polymeric nanofibers [10], collagen protein [11], and sodium alginate [12], among which CS is a cationic marine natural polysaccharide obtained from chitin after deacetylation of the *N*-acetyl glucosamine groups [13]. It is extensively applied in clinical practice due to the advantages of being self-biodegradable, biocompatible, non-antigenic, water absorbable, air and moisture permeable, non-toxic, and well adherent to skin without irritation [14]. In addition, studies in the past years have demonstrated that CS has certain antimicrobial activity, and in its dilute acid solution, free amino groups can be protonated such that CS carries positive charges. Then, the positively charged amino group can directly attack the negatively charged bacterial cell membrane via electrostatic attraction to disrupt the cell membrane to achieve a good antibacterial effect, which differs from target-specific interactions of conventional antibiotics and prevents drug resistance of bacteria from occurring to some extent [15,16,17]. By now, some CS-based wound dressing products have been developed in dosage forms of hydrogel [18,19], film [9], and sponge [20], among which hydrogel or hydrogel film is able to provide a moist environment to the wound, effectively preventing tissue dehydration and cell death, enhancing the migration of inflammatory cells and growth factors, facilitating air exchange and angiogenesis, serving as a microbial barrier, removing excessive exudate, and accelerating wound healing, among other merits, and thus has good application prospect [21,22,23].

Gentamicin (GT) is an aminoglycoside antibiotic with efficient antimicrobial activity, and, therefore, widely applied in the treatment of microbial infections, in particular, burn wounds [24]. If acting directly on skin, it will be difficult for GT to penetrate skin to deeper layers. Its systemic absorption is low probably due to its cationic nature, and therefore its efficacy mainly stems from a topical effect of the superficial layer of skin [25]. Nonetheless, GT can induce renal tubular necrosis and renal tubular congestion [26] and kill intra-auricular lymphocytes [27] to trigger certain nephrotoxicity and ototoxicity and is therefore limited in clinical application to some extent. In light of the toxic side effect of GT, many researchers treat GT by embedding [10,28] or fixing [29] to control GT release so that GT can act persistently. Alternatively, GT is delivered to a target site for specific binding to maintain prolonged action between the antibiotic and the target site by local delivery [30]. Based on this practice in combination with the above excellent properties of CS and application potential of CS gel formulation in wound repair, the present work intended to prepare a CS-GT hydrogel and further investigate its potential skin scald repair-promoting effect.

CS-GT has been successfully prepared in a previous study [31]. In this study, in order to investigate its potential scald healing-promoting effect, the antimicrobial activity of CS-GT was further studied, and the cytocompatibility and hemocompatibility of CS-GT were determined by cell viability assay and hemolysis assay.

## 2. Results and Discussion

### 2.1. Antibacterial Activity

**Figure 1:**
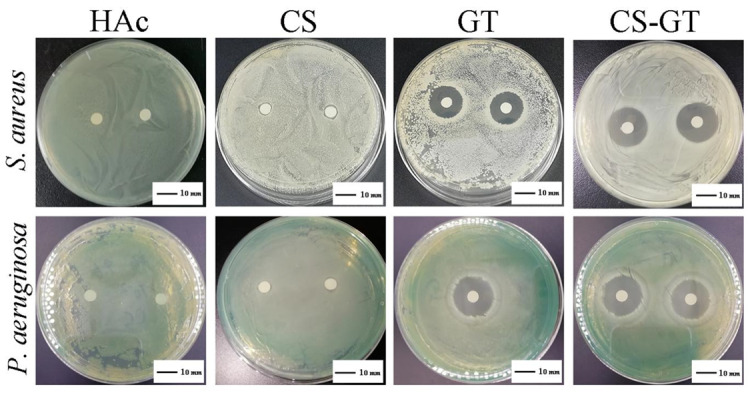
Zones of inhibition of glacial acetic acid (HAc), chitosan (CS), gentamicin (GT), and chitosan-gentamicin conjugate (CS-GT) against *S. aureus* and *P. aeruginosa*.

A desirable wound dressing should have certain antimicrobial functions to minimize pathogens to reduce inflammatory response and tissue fluid exudation at the wound site and accelerate the healing process [32]. Figure 1 shows zones of inhibition of CS, GT, and CS-GT against *S. aureus* and *P. aeruginosa*, respectively. CS samples were dissolved with 1% glacial acetic acid (HAc). As a result of adding dropwise 1% HAc alone onto paper discs (6.0 mm in diameter), zones of inhibition were all 6.0 mm in size, and, therefore, HAc solvent has no inhibitory effect against both test strains. Measurement of inhibition zones shows that diameters of the zones of inhibition of CS, GT, and CS-GT against *S. aureus* were 7.0 ± 1.0, 17.7 ± 1.2, and 20.0 ± 1.0 mm, respectively, and diameters of the zones of inhibition of CS, GT, and CS-GT against *P. aeruginosa* were 7.0 ± 1.0, 21.3 ± 0.6, and 20.3 ± 0.6 mm, respectively (Table 1). The results suggest that GT and CS-GT have comparable antibacterial activity against *P. aeruginosa*. However, these two samples have significant differences in the diameter of the zones of inhibition of *S. aureus* (*p* < 0.05). Compared with the results of CS, diameters of zones of inhibition of CS-GT against both test strains increased by 13.0 and 13.3 mm due to the introduction of GT, respectively, indicative of superior antimicrobial activity.

### 2.2. Cell Viability

**Figure 2:**
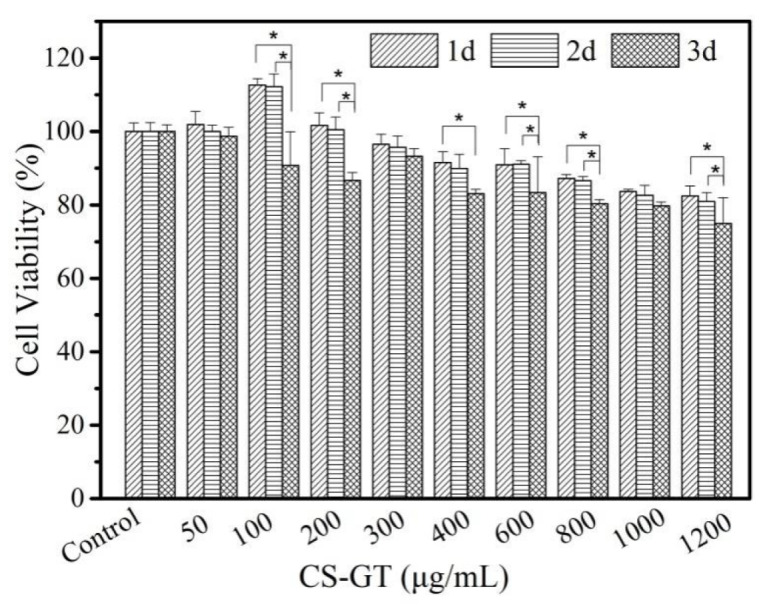
Cell viability of CS-GT (mean ± SD, * *p* < 0.05, *n* = 6).

As shown by the results of the viability assay of L929 cells in the presence of CS-GT (Figure 2), cell viability decreased gradually with the increase of CS-GT concentration. After CS-GT and L929 cells were co-incubated for 1 and 2 days, CS-GT at a concentration of 100 μg/mL promoted cell growth to some extent, and cell viability values were 112.65% and 112.23%, respectively, indicating that CS-GT at this concentration is non-toxic to L929 cells. After CS-GT and L929 cells were co-incubated for 3 days, cell viability decreased to 90.79%, indicating that CS-GT at this concentration exhibited limited cell inhibition, possibly because cell proliferation via cell division led to higher cell density and competition among cells for nutrition (*p* < 0.05 compared to days 1 and 2) [33]. For CS-GT at a concentration of 200 μg/mL, cell viability values on days 1 and 2 were 101.68% and 100.44%, respectively, and no significant impact on cell growth was noted. On day 3, cell viability decreased to 86.73%, probably due to combined action of CS-GT and cell division and proliferation (*p* < 0.05 compared to days 1 and 2). For CS-GT samples at concentrations between 300 and 1200 μg/mL, cell viability values on days 1 and 2 decreased in turn, respectively. However, at the same concentration, cell viability decreased less. On day 3, reduction in cell viability at each concentration was greater than those on days 1 and 2. At a concentration of 1200 μg/mL, cell viability decreased to 75.00%, the lowest one among cell viability values at all concentrations. According to ISO standard, after full action between a substance and cells, a concentration with cell viability above 75% is deemed not cytotoxic [34]. In summary, CS-GT samples at concentrations below 1200 μg/mL were non-toxic to L929 cells. In particular, at lower concentrations (e.g., 100 μg/mL) with action time not longer than 2 days, CS-GT promoted cell growth to some extent, indicating CS-GT has good cytocompatibility.

**Figure 3:**
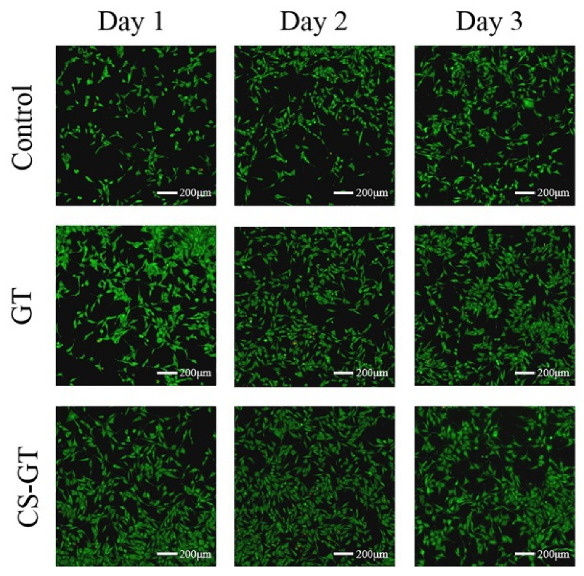
Live/dead staining analysis of cell compatibility of the sample. L929 cells in GT and CS-GT were stained after incubation for 1, 2, and 3 days.

L929 cells were treated with GT and CS-GT samples at a concentration of 100 μg/mL for 1, 2, and 3 days, respectively, and such cells were stained with AM/PI in these 3 consecutive days to observe the cell state (Figure 3). After reacting with cellular lactonase, calcein-AM emits green fluorescence and renders viable cells stained. Ethidium homodimer-1 binds to the DNA of dead or damaged cells, rendering dead cells stained red. Based on live/dead staining analysis, with the increase of culture time, a great number of normally-shaped green viable cells and a sequentially increased minor amount of red dead cells were observed in the control group. After L929 cells were treated with GT, more and more red dead cells were observed with the increase in incubation time. While the cells treated with CS-GT were basically normal in morphology, fewer red dead cells were observed, and the variability of cell staining versus time was similar to the case of the control group. Results of L929 cell viability (Figure 2) and observation of cell staining state further indicate that CS-GT is non-cytotoxic to L929 cells and has good cytocompatibility.

### 2.3. Hemolysis Assay

**Figure 4:**
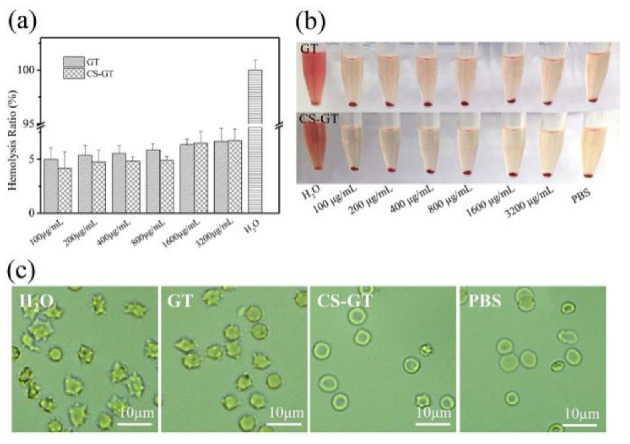
(**a**) Hemolysis rates of RBCs treated with GT and CS-GT, respectively; (**b**,**c**) images of RBCs treated with GT and CS-GT, respectively (mean ± SD, *n* = 3).

Hemolysis assay can be used to evaluate material hemocompatibility as a hemolysis rate of ≤5% meets the hemolysis criterion for biomaterials [35]. Based on the hemolysis results of GT and CS-GT in Figure 4, the hemolysis rate of GT at a concentration of 100 μg/mL was lower than 5%, while the hemolysis rate of GT at a concentration of 200 μg/mL or above was higher than 5%. The hemolysis rate of CS-GT at a concentration of 800 μg/mL or below was lower than 5% (Figure 4a), indicating that CS-GT at the concentrations below 800 μg/mL has no hemolytic activity on red blood cells (RBCs) and is more hemocompatible than GT [36]. At the same concentrations, there was no significant difference in hemolysis rates between the two groups. As shown in Figure 4b, the content of cell-released hemoglobin increased with the increase of GT and CS-GT concentrations, and the higher the content of released hemoglobin, the stronger the hemolytic activity [37]. However, in general, at a concentration below 800 μg/mL, GT induced higher hemoglobin release than CS-GT, indicating that hemolytic activity of CS-GT was lower than that of GT. In addition, in order to further assess the hemolytic properties of GT and CS-GT, the morphology of RBCs treated with GT and CS-GT at 800 μg/mL was microscopically observed, and the results are shown in Figure 4c. After treatment with water, all RBCs were disrupted basically. Additionally, after treatment with PBS, RBCs were round-shaped and basically free of damage. As compared with PBS-treated RBCs, GT-treated RBCs exhibited more prominent damage and rupture in morphology, while only a minimal part of CS-GT-treated RBCs was damaged, without significant morphological variation in general. The above results indicate unanimously that the hemocompatibility of CS-GT is superior to GT.

### 2.4. Wound Macroscopy and Healing Rates

**Figure 5:**
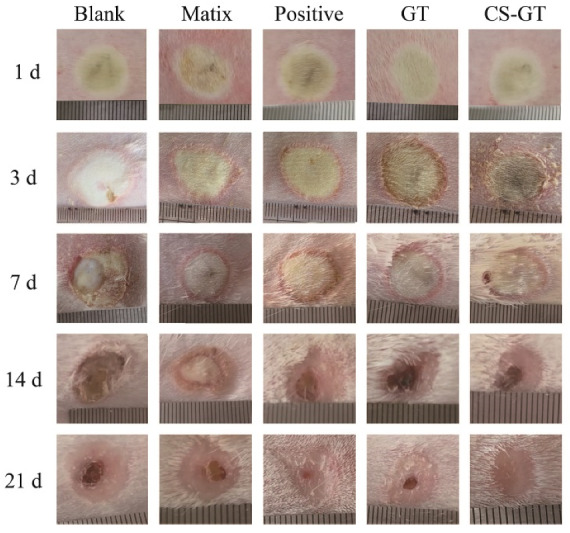
Different phases of wound healing in New Zealand rabbits treated with hydrogels.

Based on information on scald wound healing in each group at various time points in Figure 5, the scald wounds appeared subcircular and edematous, and their surfaces were softened and blanched on the day of modeling (Day 1). Over time, scald areas in each group turned smaller gradually, while the wound healing rate tended to increase (Table 2). Throughout the healing cycle, blank group and matrix group did not differ significantly in macroscopic wound change, and no significant difference in wound healing rate was noted in either group, indicating that the PVP matrix has no significant impact on wound healing. On day 7, the edema on the wound surface in each group began to resolve; however, in the case of the blank group, such edema was associated with certain tissue fluid exudation and purulent substance secretion and inflammatory response around the wound was more significant. On day 14, the peripheral scab of each scald wound in each group began to fall off, and the wound surface was dry without tissue fluid exudation and purulent substance secretion. The wound turned smaller without a distinct boundary with surrounding normal tissue, whereas effects on wound shrinkage in the positive control group, GT group, and CS-GT group were superior to those in the blank group and matrix group to a varied extent. On day 21, in the positive control group and CS-GT group, scald wound scabs basically fell off; however, a swelling phenomenon occurred in the positive control group where the scabs fell off. In the cases of the blank group, matrix group, and GT group, a minor amount of scabs were still attached to wound cores and appeared pale red.

A wound healing rate of 100% is a criterion for trauma repair. As shown in Table 2, throughout the healing cycle, the blank group and matrix group had no significant difference in wound healing rate, indicating that the matrix has no prominent action on wound healing. On day 3 after scalding, wound healing rates in the blank group were all negative, possibly because blank samples had no anti-inflammatory effect after scald wound modeling. When various proinflammatory cytokines were triggered, such wounds started to get inflamed and edematous, resulting in a larger wound area. On day 3, compared with the blank group, the GT and CS-GT groups exhibited significant wound healing effects, suggesting that GT and CS-GT exhibited stronger antimicrobial effects in the early phase of wound healing to reduce infection probability and shorten the inflammation phase so that the proliferation phase came in advance. On day 7 after trauma, wound healing rates in the blank group were the lowest, although wound healing rates in the positive control, GT, and CS-GT groups did not differ from those in the blank group significantly, which increased to a varying extent. The wound healing effects in the GT and CS-GT groups were the best, which was thought to be closely associated with the strong antibacterial activity of GT and CS-GT. On day 14, wound healing rates in the CS-GT group were 89.18% ± 11.75%, significantly different (*p* < 0.05) from those in the blank group (55.88% ± 7.07%). On day 21, in CS-GT group, the healing rates reached 99.61% ± 0.23% (*p* < 0.01 vs. blank group), and wounds were basically intact, while in the blank group, the healing rate was only 75.45% ± 2.17% and scabs did not fall off, which were congruent with the above macroscopic wound recovery. In the early phase of trauma, healing in the positive control group was slower than that in the CS-GT group. However, in the late phase of wound healing, the positive control sample had better trauma repair function, and wound healing rates on day 21 reached 94.98% ± 0.04%, only lower than those in CS-GT group. In contrast, GT had no trauma repair effect, and its healing effect in the late phase was inferior to that of CS-GT, with wound healing rates of only 87.50% ± 2.09% after 21 days of action. A synergic effect was observed to occur between CS and GT in the CS-GT group, and therefore in the late phase of trauma healing, the wound healing rates in the CS-GT group were significantly higher than those in the GT group.

### 2.5. Histological Observation

**Figure 6:**
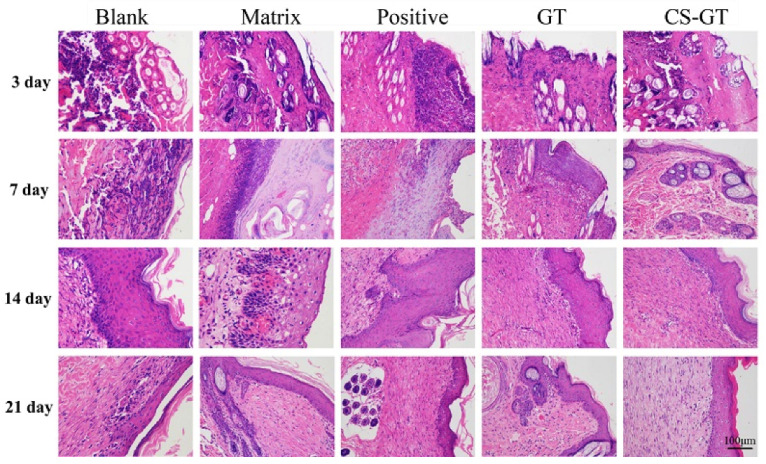
Hematoxylin and eosin (HE) staining images showing pathological changes of skin in various groups (200×).

In order to further study the effects of the hydrogel on the regeneration of wound epidermis and dermis, skins of animals in each group were sampled on days 3, 7, 14, and 21, respectively, followed by hematoxylin and eosin (HE) staining to investigate wound tissue reepithelialization. As shown in Figure 6, on day 3 after scalding, epidermal layers in each group were seriously damaged, vesicles and inflammatory cell infiltration were visible in dermal layers, and dermal interstitium became loose. However, as compared with the blank group, inflammatory cell infiltration was milder in the GT and CS-GT groups. On day 7 after scalding, edema and many inflammatory cells were still present in the blank and matrix groups, and fewer inflammatory cells were observed in the positive control group. In contrast, no epithelial regeneration was observed in all three groups. The inflammatory cell infiltration in the GT group was the mildest, whereas that in the CS-GT group was worse. In both groups, epithelial regeneration was observed. On day 14 after scalding, in addition to mild inflammatory cells, hyperkeratosis was also observed in the blank, matrix, and positive groups, while in the GT and CS-GT groups, complete epidermal regeneration was observed. On day 21 after scalding, scabs did not fall off completely, particularly in the blank group, and epidermal regeneration and angiogenesis, to some extent, were observed in other groups, especially in the CS-GT group, where connective tissue recombination was the best and previously unordered tight structure became more regular. In general, the skin healing process in the CS-GT hydrogel group was basically completed on day 21. The animals in CS-GT group had the thickest dermis, dense dermal mesenchyme, and spindle-shaped fibroblasts, indicative of optimal recovery.

**Figure 7:**
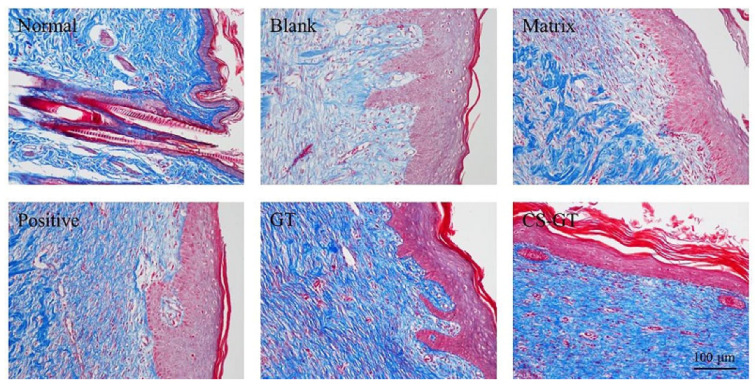
Masson staining on Day 21 postinjury (200×).

The final tissue remodeling phase of trauma repair is tissue maturation and extracellular matrix deposition, mainly manifested as a gradual increase and recombination of collagen fibers [38]. Masson staining is classical staining of collagen fibers; in this study, skins at original scald sites in each group were sampled on day 21 for Masson staining to check collagen content wherein. As shown in Figure 7, compared with normal skin, the skin in the blank and matrix groups had fewer stained collagen fibers, which were sparsely and unorderly arranged. In contrast, in the positive control, GT, and CS-GT groups, the content of stained collagen in the skin increased significantly. Additionally, in the case of the CS-GT group, collagen fibers in the skin were orderly arranged with more uniform density.

**Figure 8:**
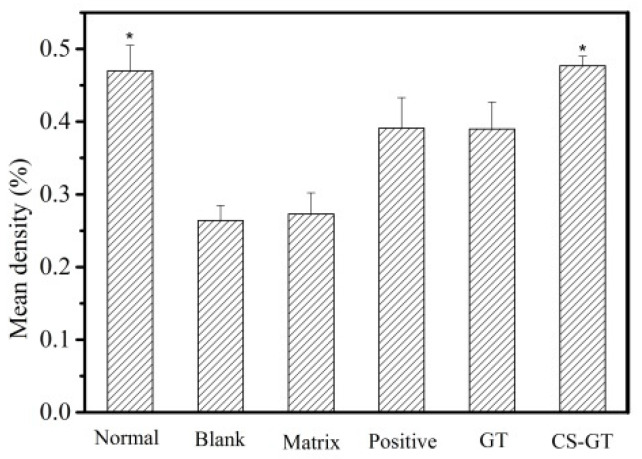
Mean density values of collagen in scald wound in various groups (mean ± SD, *n* = 4). Note: versus blank control group, * *p* < 0.05.

In addition, Image-pro plus software was used to quantitate the collagen fiber content (expressed as mean optical density) in a Masson plot. As shown in Figure 8, the collagen fiber contents of skin in the matrix group were comparable to those in the blank group, indicating that the PVP matrix has no significant impact on collagen fibrogenesis in the scald-skin healing process. In CS-GT group, the collagen fiber content in the skin was close to the level in normal skin, which was significantly different from those in the blank group (*p* < 0.05). The results of the positive control and GT groups were intermediate between those of the blank group and normal skin. Therefore, it can be found that the scald-skin healing effect in the CS-GT group was superior and the efficacy was most significant.

### 2.6. TP and HYP Contents in Granulation Tissue

**Figure 9:**
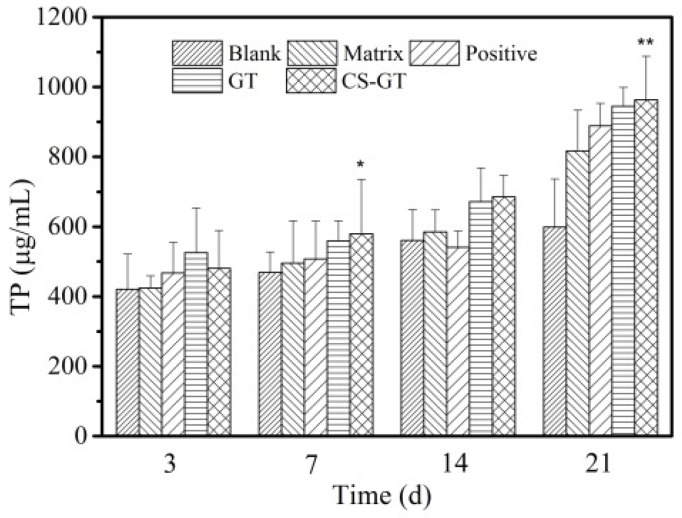
Total protein (TP) contents of wounds in various groups (mean ± SD, *n* = 4). Note: versus blank control group, * *p* < 0.05, ** *p* < 0.01.

After a skin trauma, protein in granulation tissue will be substantially hydrolyzed, enabling the growth of a great number of bacteria at the wound, which will affect the epidermal formation and further wound healing rate. TP is the content of all proteins during the growth of granulation tissue on the wound surface. As shown in Figure 9, the content of TP in skin wound granulation tissue in each group increased gradually over time. Throughout the study cycle, the TP level in the skin in the blank group was basically consistent with that in the matrix group, without significant difference, indicating that PVP has no significant impact on TP content in tissue. On day 3 after scalding, compared with the TP content in the blank group, the TP contents in the positive control, GT, and CS-GT groups increased to some extent, among which the GT group had the highest TP content. On day 7, there was a trend similar to that on day 3. However, for the CS-GT hydrogel group relative to that in the blank group, the increase in TP content was significantly different (*p* < 0.05). From days 14 to 21, variations in TP content in the blank group were smaller, whereas an increase in TP in other groups was significant. On day 21, the TP content in CS-GT group was the highest with a significant difference from that in the blank group (*p* < 0.01). In summary, compared with GT alone, CS-GT was more capable of accelerating protein synthesis in wound granulation tissue.

**Figure 10:**
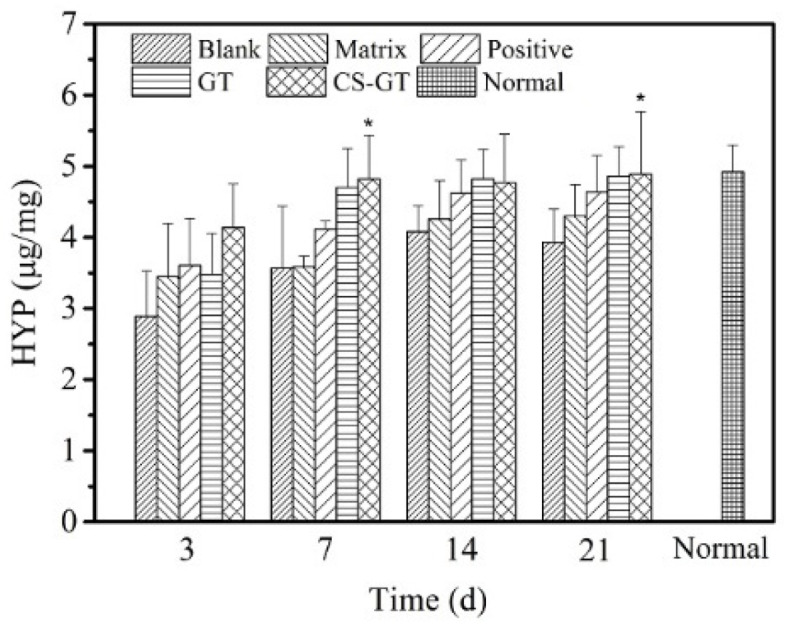
Hydroxyproline (HYP) contents of wounds in various groups (mean ± SD, *n* = 4). Note: versus blank control group, * *p* < 0.05.

During wound healing, connective tissue mainly consists of fibroblasts and collagen, and collagen content has an immediate impact on the wound repair process. HYP is a precursor amino acid of collagen synthesis and the HYP content of granulation tissue represents the collagen level; therefore, a study on HYP content enables further reflection of wound healing [39]. Based on variations in HYP content in various groups (Figure 10), on day 3 after scalding, as compared with normal skin, scalding led to a significant decrease in HYP content in skin wound tissue. From days 3 to 21, HYP content in wound tissue in each group increased gradually, among which, HYP content in CS-GT group at various time intervals was higher than those in the blank, matrix, and positive groups. The HYP content in the CS-GT group on days 7 and 21 exhibited significant differences from that in the blank group (*p* < 0.05). Additionally, from day 7 on, HYP contents in both the GT and CS-GT groups were close to that in normal skin. The above findings indicate that CS-GT hydrogel is able to effectively promote HYP synthesis in traumatic tissue and accelerate collagen synthesis and accumulation so as to provide a material basis for expediting wound healing.

### 2.7. Expression of Proinflammatory Factors

After skin damage, the damaged tissue will undergo a slow-onset persistent inflammatory response focusing on the release of pro-inflammatory factors TNF-α and IL-6, among which, TNF-α is capable of stimulating vascular endothelial cells to regulate cell metabolism within an organism [40], while IL-6 has an immediate impact on the growth of fibroblast and endothelial cells [41]. However, the persistent presence of excessively high levels of TNF-α and IL-6 in tissues would enhance the toxicity of inflammatory cells and induction capacity of related inflammatory mediators, and eventually, leading to damage and necrosis of more tissue cells [42].

**Figure 11:**
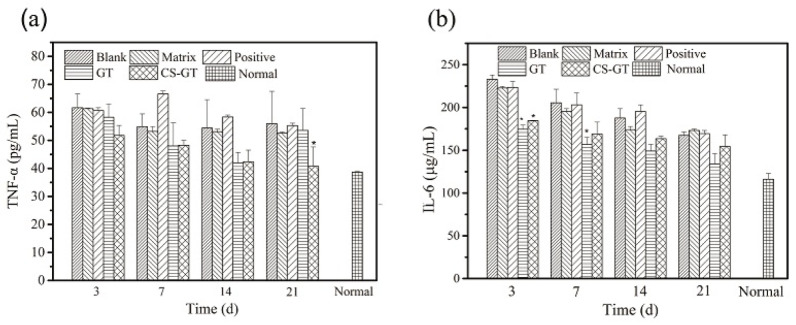
Levels of TNF-α (**a**) and IL-6 (**b**) in wounds in various groups (mean ± SD, *n* = 4). Note: versus blank control group, * *p* < 0.05.

Based on the levels of TNF-α and IL-6 in wound tissue in various groups (Figure 11), throughout wound healing, the levels of TNF-α and IL-6 in skin tissue in the blank group did not differ significantly from those in matrix group, indicating that PVP matrix has no significant impact on the inflammatory response of wound tissue. From days 3 to 21 after scalding, the IL-6 levels in wound tissue in various groups tended largely to decrease gradually over time and were all higher than the level (Normal) in normal skin. In particular, in the early phase (days 3 and 7), the IL-6 levels in the skin in the GT and CS-GT groups decreased significantly, suggesting that the test article has a good inhibitory effect against early inflammation. On day 21, only during the study cycle, the TNF-α level in the CS-GT group was significantly lower than that in the blank group and close to the level in normal skin, and such levels in all the other groups did not differ significantly from the level in the blank group. Therefore, CS-GT hydrogel is superior to that of positive control, and GT can alleviate the local inflammatory response of a wound to accelerate scald wound healing.

## 3. Materials and Methods 

### 3.1. Experimental Materials

Chitosan (544kDa, degree of deacetylation 86.7%, Nantong Xingcheng Biological Products Factory, Qingdao, China), gentamicin sulfate (AR, Aladdin Reagent Company, Shanghai, China), polyvinylpyrrolidone (medical grade, Shanghai Aladdin Reagent Company, Shanghai, China), wet burn ointment (Shantou Meibao Pharmaceutical Co., Ltd., Shantou, China), Interleukin IL-6 ELISA Kit (rabbit-IL-6, Nanjing Jiancheng Biotechnology Research Institute, Nanjing, China), Tumor Necrosis Factor TNF-α ELISA Kit (rabbit-TNF, Nanjing Jiancheng Biotechnology Research Institute, Nanjing, China), TP Kit (A045-3, Nanjing Jiancheng Bioengineering Research Institute, Nanjing, China), HYP Kit ( A030-2, Nanjing Jiancheng Bioengineering Research Institute, Nanjing, China), and Calcein-AM/PI Live Cell/Dead Cell Double Staining Kit (500T, Shanghai Ye Sheng Biotechnology Co., Ltd., Shanghai, China).

Benchtop super temperature control scalder (YSL-5Q, Beijing ZhongshiDichuang Technology Development Co., Ltd., Beijing, China), microplate reader (DNM-9602, Beijing Prolong New Technology Co., Ltd., Beijing, China), inverted microscope (ECLIPSE Ti, Nikon, Tokyo, Japan), and portable high-speed disperser (S10, Ningbo Xinzhi Co., Ltd., Ningbo, China).

### 3.2. Experimental Materials

Sixteen New Zealand rabbits (half male and half female, conventional grade) weighing 2000 ± 20 g before modeling were provided by Guangdong Medical Laboratory Animal Center. The laboratory animal production license number is SCXK (Guangdong) 2014-0035, and the laboratory animal quality certificate number is 44411000004907. The rabbits were individually housed with free access to water and food under the following conditions: 25 °C temperature, 40–70% humidity, and 12 h light/12 h dark cycles. Experimental study on scald repair of CS-GT hydrogels after one week of adaptive breeding. Animal experiments were undertaken according to guidelines set by the Experimental Animal Center of Guangdong Ocean University (Guangdong, China) for the care and use of laboratory animals (SYXK (Yue) 2014-0053). This study was approved by the Animal Ethics Committee of Guangdong Ocean University. 

### 3.3. Preparation of CS-GT Hydrogel

To fabricate CS-GT hydrogel, 8 g of PVP was firstly dissolved in 32 g of distilled water, which was then mixed with 20 g CS-GT aqueous solution containing 5 g of CS-GT [31] and 40 g glycerin under magnetic stirring till a homogeneous solution was obtained. GT hydrogel was prepared using the same method, where 5 g GT was introduced to replace the 5 g CS-GT. For comparison, the matrix was also prepared based on the same procedures without CS-GT and GT loaded.

### 3.4. In Vitro Antibacterial Assay

As *S. aureus* and *P. aeruginosa* often exist in patient exudate and are important pathogenic strains causing infections in burn/scald patients [43]. These two strains were chosen in this study as indicator microorganisms, followed by antimicrobial assay of CS-GT via diameter of zone of inhibition [44] according to the following procedure: 100 μL of bacterial suspension (1 × 10^8^ CFU/mL) was evenly plated onto NB agar plate, and a sterile paper disc (6.0 mm in diameter, sterilized by autoclaving) was placed on the agar surface, onto which a test sample was then added dropwise. After the sample was incubated at 37 °C for 24 h, the diameter of the zone of inhibition was determined. The solvent of 1% HAc, CS-GT, and GT was distilled water, while the solvent of CS was 1% HAc. The concentration of all samples was 1 mg/mL. GT’s graft rate was 22.1%, as reported in our previous research [31].

### 3.5. In Vitro Cytotoxicity Study

In this study, MTT assay was used to determine the effect of CS-GT on the viability of human skin fibroblasts (L929) so as to evaluate it in vitro cytotoxicity. A normal control group (Control) and test groups of CS-GT at various concentrations (100, 200, 300, 400, 600, 800, 1000, and 1200 µg/mL, respectively) were set up. Cells in good growth state were selected, and after adjustment of cell concentration, they were seeded onto a 96-well culture plate at a plating density of 5 × 10^3^ cells/well (100 μL per well, 6 replicate wells per group); then, the plate was put into a cell incubator (37 °C, 5% CO_2_) and incubated for 24 h. After the culture medium was pipetted off, blank medium and DMEM medium for CS-GT at varied concentrations were added to the wells (100 μL/well), respectively, and the plate was put into the incubator again for further culturing. Cell viability assay was performed for each group by MTT after every 1 day of culture for 3 consecutive days to study the effects of CS-GT in varying action time on L929 viability [45]. The absorbance at 570 nm was measured by an RT-2100C microplate reader.

Cell viability was calculated using the following formula:

$$\mathrm{Cell}\;\mathrm{viability}\left(\;\%\right)\;=\;\frac{{\mathrm{OD}}_{\mathrm{test}\;\mathrm{well}}\;-\;{\mathrm{OD}}_{\mathrm{blank}\;\mathrm{well}}}{{\mathrm{OD}}_{\mathrm{control}\;\mathrm{well}}\;-\;{\mathrm{OD}}_{\mathrm{blank}\;\mathrm{well}}}\;\times \;100\%$$

In addition, in order to have a more straightforward observation of the compatibility of the test sample with L929 cells, the cells in this study were stained using a Calcein-AM/PI Double Stain Kit to observe cell viability state [46]. Cell suspension was prepared with 1× assay buffer with density ranging from (1 × 10^5^) to (1 × 10^6^) cells/mL. Then, such cell suspension was divided into two aliquots, into which CS-GT (100 µg/mL, 50 µL) and GT (100 µg/mL, 50 µL) were added, respectively, followed by incubation for 24, 48, and 72 h, respectively. Then, 100 µL of staining working solution was added into 200 µL of each part of cell suspension, mixed well, incubated at 37 °C for 15 min, and stained; then, cell morphology and staining status were observed using an inverted fluorescence microscope.

### 3.6. Hemolysis Assay

According to the literature [36], the hemolysis rate of CS-GT was determined as follows: 3 mL of rat blood was centrifuged at 2000 rpm for 15 min to isolate RBCs from serum, and then the above RBC sediment was washed with 1× PB buffer three times until a clear supernatant was observed. Next, the RBC sediment was diluted with PBS to a concentration of 2% (*v/v*) for later use, while RBCs incubated with deionized water and PBS were used as the positive and negative controls, respectively. GT and CS-GT solutions at various concentrations (100, 200, 400, 800, 1600, and 3200 μg/mL) were added into the above 2% RBC suspension, respectively. RBS suspension samples prepared as above were incubated at 37 °C for 1 h and then centrifuged at 2000 rpm for 15 min; the image of each centrifuged sample was captured with a digital camera, and absorbance of each supernatant was measured at 540 nm with a plate reader. The hemolysis rates were calculated using the following formula:

$$H\%\;=\;\frac{H_{1}\;-\;H_{0}}{H_{100}\;-\;H_{0}}\;\times \;100\%$$

where *H_0_*, *H_1_*, and *H_100_* are absorbance values of the negative control (PBS), test sample, and positive control (H_2_O), respectively.

In addition, GT-treated RBCs and CS-GT-treated RBCs at a concentration of 800 μg/mL were microscopically examined as follows for any morphological change: After incubation at 37 °C for 1 h, the RBC solutions were centrifuged at 20,000 rpm for 20 min. The supernatants were photographed using a digital camera. The collected RBC pellets were diluted in PBS, dispensed onto clean glass slides, covered with a coverslip, and then photographed by using a microscope equipped with a digital camera.

### 3.7. In Vivo Animal Test

**Figure 12:**
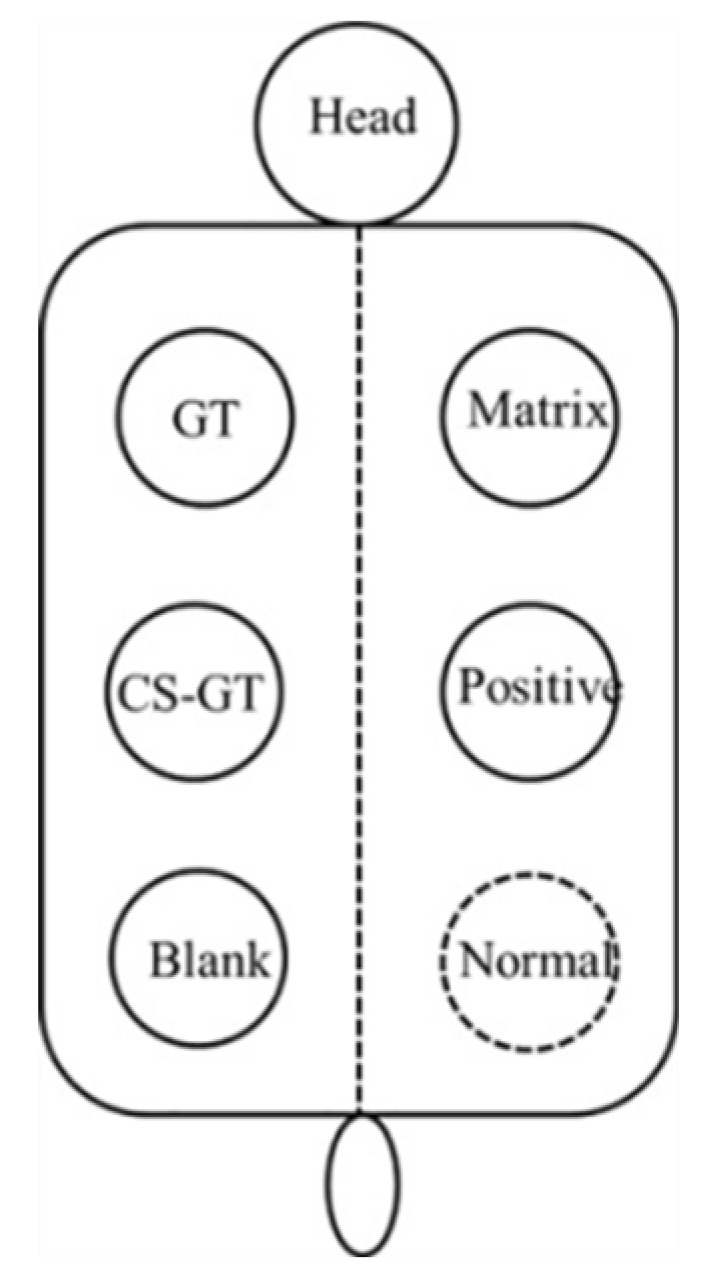
Correspondence of groups to scald wounds.

Twenty-four hours before the experiment, each New Zealand rabbit was dehaired using 10% sodium sulfide, then, the dehaired area was immediately cleaned with lukewarm water and the rabbits housed in a single cage. Briefly, 3% pentobarbital sodium (30 mg/kg) was injected via ear vein to anesthetize the New Zealand rabbit and 75% ethanol was used to disinfect the skin of dorsal hair loss area, then, 5 scald wounds of 1.5 cm^2^ in area were created laterally at points 2 cm away from the dorsal midline of the rabbit by using a scalding apparatus. The scalding conditions were described as follows: probe temperature, 100 °C; working pressure, 1000 g; probe-to-skin contact time, 6 s. Each animal was self-controlled, and 5 wounds of each rabbit correspond to the blank group, matrix group, positive control group, GT group, and CS-GT group, respectively, as shown in Figure 12.

After modeling, scald wounds exhibited typical scald features: pale opalescent appearance and dry surface. In the case of the blank group, no treatment was performed after successful modeling. In the case of matrix group, the scald was daily treated by applying PVP hydrogel, once in the morning and once in the evening (about 1 g/dose) for 21 consecutive days starting on day 1 after modeling. In the cases of the positive control, GT, and CS-GT groups, moist exposed burn repair ointment (MEBO), GT hydrogel, and CS-GT hydrogel were applied, respectively, in the same manner as in the case of the matrix group. On the day of modeling and upon dressing change each time, size, color, exudate, scab formation, and scab falloff of each wound were inspected, photographed, and documented. On days 3, 7, 14, and 21, variations in wound area were determined as follows to evaluate wound healing: wound closure was monitored by imaging with a digital camera and a scale bar, and wound area was quantitated by Photoshop so that pixel area was calculated from wound contour plotted by a blinded observer. Then, wound healing rate *M* was calculated using the following formula:

$$M\%=\frac{M_{0}\;-\;M_{1}}{M_{0}}\times 100\%$$

where *M_0_* is the initial wound area, and *M_1_* is wound area at a time point.

### 3.8. Histological Observation

In histological examination, on days 3, 7, 14, and 21 after scalding, 4 test animals in each group were euthanized, respectively, and a full layer of skin tissue of about 1.5 cm^2^ in area around each original scald site was immediately incised as a sample. A small patch of skin sample incised from the center of the scald was fixed in 4% paraformaldehyde (PFA) for histology and collagen deposition examination (Masson staining method), while the remaining skin tissue samples were used for subsequent monitoring of biochemical parameters. The above tissue samples, which had been fixed with 4% PFA for 24 h, were embedded in paraffin and then cut with paraffin microtome (CUT 4050, Micro Tec, Inc., Walldorf, Germany) in a direction longitudinal to the tissue, followed by staining with HE and modified Masson trichrome staining kit (Beijing Leagene Biotechnology Co., Ltd., Beijing, China), respectively. Thereafter, the regeneration of epidermis and dermis, and the level and distribution of collagen fibers in each skin sample were examined via an optical microscope (DMI3000B, Leica).

### 3.9. Determination of TP Content and Proinflammatory Cytokine Levels

The desired amount of each remaining skin tissue sample was accurately weighed, mixed with normal saline at a ratio of weight (g): volume (mL) = 1:9, and mechanically homogenized in an ice water bath. The resulting homogenate was cryogenically centrifuged to isolate supernatant, and content of TP and levels of proinflammatory cytokines (TNF-α and IL-6) in tissue in the supernatant were determined as per kit operating procedure.

### 3.10. Determination of HYP

Take an appropriate amount of the remaining fresh skin tissue samples from each of the above groups, accurately weighed them, and then measured the content of HYP according to the kit method.

### 3.11. Statistical Analysis

The data were processed with SPSS 17.0 software and were analyzed using independent-samples *t*-tests. Numerical data are expressed as means ± SD, and the differences among groups were analyzed using a one-way analysis of variance. *P* < 0.05 was used to indicate statistical significance.

## 4. Conclusions

This study demonstrates that CS-GT has efficient antimicrobial properties, as evidenced by viability assay and hemolysis assay of L929 cells that CS-GT is not cytotoxic and has good cytocompatibility and hemocompatibility. With CS-GT as an active repair component and PVP as the matrix, CS-GT hydrogel was prepared. The application of this hydrogel enabled quicker scald healing and shorter healing time in animals. Biochemical analysis shows that by facilitating the synthesis of TP and HYP in granulation tissue, CS-GT hydrogel promoted collagen fibrosis while reducing cytokine levels in an inflammatory response, and thereby accelerated wound healing. All these results demonstrate that CS-GT hydrogel is a promising efficient novel scald repair dressing.

## Figures and Tables

**Table 1:** Diameters of zones of inhibition in various groups (unit: mm, mean ± SD, *n* = 3). Note: versus CS group, * *p* < 0.01; versus GT group, ^#^ *p* < 0.05.

Test strain	CS	GT	CS-GT
*S. aureus*	7.0 ± 1.0	17.7 ± 1.2 *	20.0 ± 1.0 *^,^^#^
*P. aeruginosa*	7.0 ± 1.0	21.3 ± 0.6 *	20.3 ± 0.6 *

**Table 2:** Wound healing rates (%) at different times in each group of scald wounds (mean ± SD, *n* = 4). Note: versus blank control group, * *p* < 0.05, ** *p* < 0.01.

Time/d	Blank	Matrix	Positive	GT	CS-GT
3	−6.67 ± 9.31	−1.81 ± 7.78	3.72 ± 2.10	20.12 ± 7.46 **	15.10 ± 8.82 *
7	11.88 ± 6.63	19.15 ± 10.03	24.68 ± 12.92	39.7 ± 5.24	31.57 ± 12.02
14	55.88 ± 7.07	58.26 ± 6.99	76.86 ± 6.34	66.24 ± 8.59	89.18 ± 11.75 *
21	75.45 ± 2.17	77.39 ± 0.67	94.98 ± 0.40*	87.50 ± 2.09 *	99.61 ± 0.23 **
